# Comparing oculomotor efficiency and visual attention between drivers and non-drivers through the Adult Developmental Eye Movement (ADEM) test: A visual-verbal test

**DOI:** 10.1371/journal.pone.0246606

**Published:** 2021-02-05

**Authors:** Andrés Gené-Sampedro, Francisco Alonso, Celia Sánchez-Ramos, Sergio A. Useche

**Affiliations:** 1 Department of Optics and Optometry and Vision Sciences, University of Valencia, Valencia, Spain; 2 DATS (Development and Advising in Traffic Safety) Research Group, INTRAS (Research Institute on Traffic and Road Safety), University of Valencia, Valencia, Spain; 3 Faculty of Optics and Optometry, Complutense University of Madrid, Madrid, Spain; Monash University, AUSTRALIA

## Abstract

**Objective:**

The objective of this study was to assess and compare drivers’ and non-drivers’ outcomes in the Adult Developmental Eye Movement test (ADEM), a visual-verbal test that measures the time needed to read series of numbers in both a vertical and horizontal reading pattern. A set of driving parameters (i.e., experience, risk exposure, and day and night perceived difficulty) and demographic variables (i.e., age, gender, and academic level) were considered as potential predictors of the test performance.

**Methods:**

For this cross-sectional study, 302 healthy subjects (age range 20 to 86 years old) completed a self-reported questionnaire aimed at retrieving data on the independent variables, and underwent the ADEM in order to obtain the dependent outcomes. 214 (70.9%) of the participants were drivers. Non-parametric analyses and multilevel linear regression were used to assess differences between the variables and a prediction model. Also, some correlations were evaluated through the Spearman test.

**Results:**

Drivers showed significantly better test performance than non-drivers. The age, driving experience, and perceived difficulty in driving at night were obtained as potential predictors of the test performance with the applied linear regression model.

**Conclusion:**

The ADEM may be a practical, non-expensive, easy-to-apply tool in the assessment of drivers, useful for obtaining or renewing the driving license. This test may help in the detection of impairments in the saccadic efficiency that could have a detrimental effect on the driving performance.

## Introduction

Traffic safety is a complex and dynamic process that impacts the public health of modern societies; also, it is highly dependent on human factors. Both physical and mental health have shown to be great contributors to driving performances [1,2]. Safe driving results from the interaction between cognitive, visual, and motor capacities of the driver, together with the car and the environment.

When driving, most of the information is received through the visual system [3–5]. By means of optimal eye movements and attention, visual information can be recognized, analyzed, and processed, thus allowing the driver to understand, organize and act within a dynamic environment [3,6–8]. In fact, visual attention errors have been related to a large proportion of traffic accidents [9–11]. Also, a significant amount of research has been published on the significance of ocular movements in real driving experiments and driving simulations [1,9,12,13]. The most important eye movements for driving are fixations, pursuits, and saccades [3–5,7,9]. Fixation mechanisms allow the person to focus on an object with the central vision and perceive details at the same time. Pursuits allow the individual to follow an object and get the maximum quantity of details within a moving environment. And, finally, saccades are the fastest eye movements, that help focus the attention on a new object appearing in the peripheral field of vision. Saccades move the eye to fix an object inside the fovea (central vision). They are highly related to the interaction between central and peripheral vision, and they have been identified as one of the most important ocular movements in both driving and other daily activities [6,8,14]. For instance, the carryover of eye-movements from one task affects the visual scanning in a second task [15,16]. Also, recent evidence has shown how ocular patterns may be responsible for a considerable proportion of human factor-related driving crashes [3,17]. Indeed, measuring saccadic eye movements, altogether with other visual function parameters, play an important role in identifying impairments in the oculomotor function and other parameters related to activities of everyday life, such as driving, and thus may be a useful tool for assessing someone’s fitness to drive [14,18].

There is no reference or golden standard to measure saccadic eye movements. The state-of-the-art methodologies include sophisticated devices that track eye movements and directly measure their basic components (e.g., velocity, accuracy, or latency) by means of helmets, glasses, or independently installed devices without physical contact with the person to be tested [9]. In addition, there are inexpensive, easy-to-conduct, visual-verbal tests such as the Developmental Eye Movement test (DEM) [19] and its version for adults, the Adult Developmental Eye Movement test (ADEM) [20], that were designed for a simple clinical testing of saccadic performance in children (DEM) and adults (ADEM) through rapid number-naming [14]. A former investigation pointed out the importance of analyzing the association of the DEM and/or ADEM with other everyday activities such as driving [14].

Both the DEM and ADEM require the subject to use visual (central and peripheral) and cognitive (automaticity of number naming) attention and processing, as well as both vertical and horizontal saccades, to read series of numbers as fast as possible (see “ADEM” section for further information) [14,20], as similarly reported in a study consisting of letter-searching (vertical and horizontal) and a picture-based road-hazard-detection task [15,16]. In this regard, these tests may be requiring (as in usual reading) the use of overt (with fixations) and covert (without fixations) attention in order to adequately read the numbers [21,22]. The DEM and ADEM have been reported to assess what Powell et al. [14] called saccadic efficiency (i.e., indirect evaluation of saccadic function in combined tracking and cognitive visual-verbal identification), rather than directly measuring the eye movements. However, there is still some controversy on whether the DEM estimates the quality of saccades or only the reading performance [23,24], with a recent study stating that the DEM test could replace an eye-tracker examination [24]. It should be also borne in mind that previous expert literature has found a learning effect within the use of the DEM, while, on the other hand, no studies have assessed this for the ADEM; however, good-to-high-reliability values have been reported and the test has been identified as suitable to be used in clinical practice [25]. Also in this regard, the correlation between both tests was found to be only moderate (*r* = .42) in one study, and thus they cannot be used interchangeably [14]. The ADEM uses two-digit numbers to compensate for the cognitive development in adults compared to children [20]. This may result in an increment in the spatial-load factor (i.e., demand on the visual system to process information about the relative position and orientation of stimuli [26]) and thus reflect a more precise estimation of saccadic eye movement, if compared to the DEM [14]. To the best of our knowledge, no previous research has investigated the potential relationship between ADEM and driving parameters.

Regarding eyesight in driving, the legislation applicable in most countries only requires primary eye health examinations to obtain or renew a driving license, with visual acuity being the most common functional method for determining a driver’s eligibility for the licensure processes [10]. In this regard, and although visual acuity plays an important role in safe driving, there are other relevant matters related to the driving task, such as eye movements, visual attentional issues, and fatigue, which may also explain a substantial part of the human-factor related traffic causalities occurring every day [10,15,16,27,28]. Thus, other types of visual testing, which can provide better information concerning the visual functional capabilities of drivers, should be added up to current examination standards [3,7]. In the light of these statements, the question arises whether the specific performance in the ADEM may be related to certain driving parameters, and thus be of interest in the visual assessment of drivers.

In addition to all the aforementioned, it is important to bear in mind the relationship between visual function, human development stages, and driving habits [10,27,29]. Also, there are sociodemographic and human factors that can influence visual health, and therefore be crucial for safe driving. For instance, female drivers are more likely than males to drive safely [13] but have a higher prevalence of visual issues [10]. Previous research suggests that other sociodemographic factors, such as the academic level, are related to the driving behavior [30,31]. Thus, the question of whether the educational level may influence visual factors related to driving comes naturally. In addition, age is another one of the most important factors that can influence visual health, and therefore road safety, due to the impairment of the driving abilities that go with age-related physiological and psychological deterioration [2,10,32–34]. Accordingly, older drivers are more affected by distractors, exhibit smaller saccades and other visuomotor impairments [4], and in general, perceive the road as more hazardous [35].

### Objectives and hypotheses

The objective of this study was to assess and compare drivers and non-drivers’ outcomes in the Adult Developmental Eye Movement test, considering a set of driving parameters (i.e, experience, risk exposure, and day and night difficulty) and demographic variables (i.e., age, gender, and academic level) as potential predictors of the test performance.

Regarding the hypotheses of the study and bearing in mind the aforementioned research supporting the influence of the study’s variables, we expected to find that: *1*) drivers will have a better test performance than non-drivers, and *2*) driving (experience, risk exposure, and day and night perceived difficulty) and sociodemographic (age, gender, and academic level) parameters will act as potential predictors of the ADEM outcomes.

## Methods and materials

### Sample

A sample size of 233 participants was determined by a power analysis (G*Power 3.0; [36]) assuming an α = .05, a power level of .95, and effect size (*f*^2^) of .075. Nonetheless, a bigger sample was selected in order to adjust to the percentages of drivers and non-drivers as presented in the Spanish Census of Drivers (72.1% drivers, 58.0% men) [37].

Thus, 302 (143 men, 159 women) healthy Spaniards with an age ranging from 20 to 86 years old were selected. Participants self-reported 2.8±0.7, 2.7±0.8, and 2.7±0.8 points in three scales measuring (in a range from 0 to 4 points) their quality of life, visual quality, and reading quality, respectively (see “Main questionnaire” section). In this regard, 68.1%, 59.3%, and 64.9% of the participants perceived excellent levels in their quality of life, eyesight, and reading ability, respectively. The mean result in the visual function test (VF-14; see “Main questionnaire” section) was 96.0±7.1 out of 100 possible points. 214 (70.9%) participants were drivers, of which 127 (59.3%) were men. All had a driving license and at least 1.5 years of driving experience (for this reason, the minimum age is set to 20 years).

**Fig 1:**
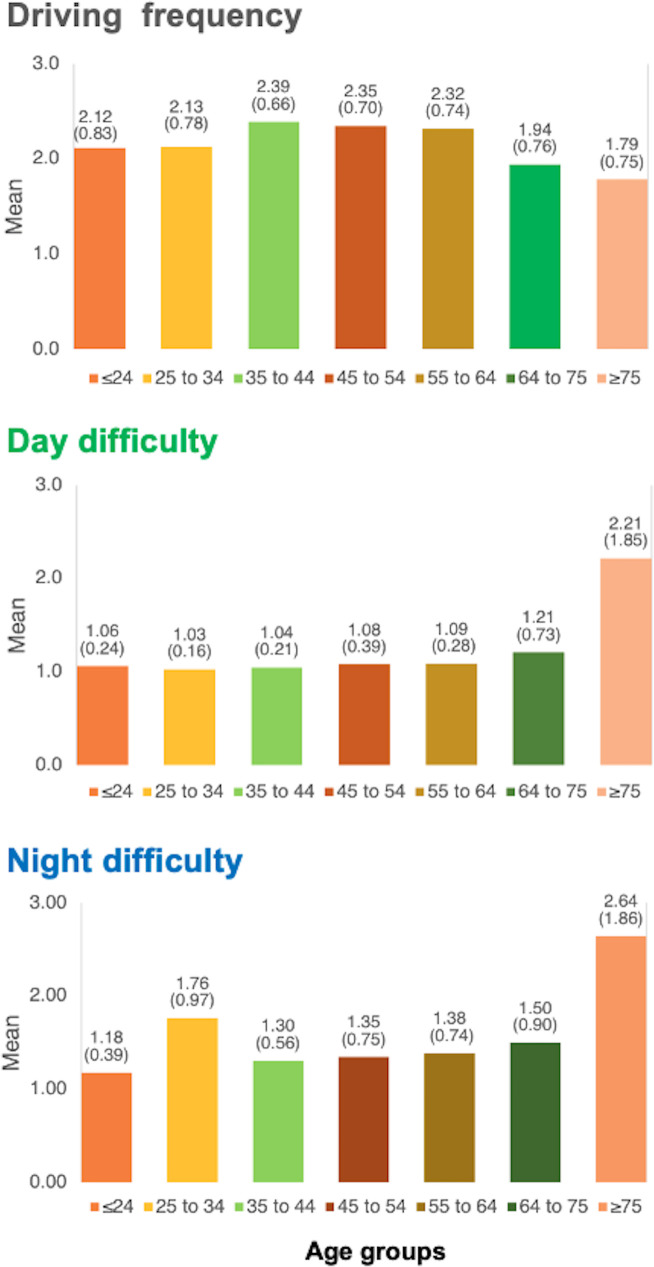
Driving frequency (top chart), perceived difficulty in driving during the day (middle chart) and night (bottom chart). Data are presented as the mean and standard deviation between parentheses.

Further relevant data on the sociodemographic (age, gender, educational level) and driving (frequency, day-driving difficulty, night-driving difficulty) features of the sample are described in detail in Table 1 and Fig 1. The general sample was divided into seven age groups (from 20 to 24, from 25 to 34, from 35 to 44, from 45 to 54, from 55 to 64, from 64 to 75 and older than 75) to facilitate the analyses of the data. In the same way, and in order to perform driving experience-based comparisons, the sample of drivers was subdivided into low-, medium-, and high-experienced (see “Main questionnaire” section).

**Table 1:** Sociodemographic variables and functional features of each one of the seven age groups.

Age group	Age Mean (SD)	Gender		Educational level			Status	
		Male	Female	Basic level	Medium level	High level	Driver	Non-driver
≤ 24 n = 26	22.2 (1.0)	34.6%	65.4%	3.1%	25.0%	71.9%	80.8%	19.2%
25–34 n = 55	29.5 (2.9)	41.8%	58.2%	18.2%	20.0%	61.8%	78.2%	21.8%
35–44 n = 31	40.5 (2.9)	45.2%	54.8%	32.1%	39.3%	28.6%	90.3%	9.7%
45–54 n = 32	50.3 (2.6)	50.0%	50.0%	28.1%	37.5%	34.4%	84.4%	15.6%
55–64 n = 65	59.9 (3.0)	49.2%	50.8%	41.5%	46.2%	12.3%	72.3%	27.7%
65–74 n = 61	69.5 (3.2)	50.8%	49.2%	55.7%	13.1%	31.1%	55.7%	44.3%
≥ 75 n = 32	80.0 (3.3)	56.2%	43.8%	56.3%	15.6%	28.1%	43.8%	56.2%
Total N = 302	52.2 (18.7)	47.4%	52.6%	37.1%	26.8%	36.1%	70.9%	29.1%

### Study design and procedure

For this cross-sectional, observational, double-blinded study, participants were randomly recruited from a traffic psychological assessment center (Red Cross Valencia) and an optometric clinic in Valencia (Spain) through a convenience (non-probabilistic) sampling method [38]. Also, neither clinical subjects nor drivers were randomly chosen from any other different sources. All measurements were carried out in the abovementioned centers under the same environmental conditions, and by the same trained optometrists and ophthalmologists (ratio researchers-subjects was always at least 1:1). All subjects were informed about the aims and procedures of the investigation protocol.

All participants underwent a full optometric examination, performed the clock-drawing cognitive test [39], and a validated version of the Mini-Mental State Examination (MMSE) for the Spanish population [40,41] to rule out possible visual and cognitive impairment. All tests were carried out binocularly and using optical correction if normally used (e.g., glasses or contact lenses). The participants also completed a questionnaire on sociodemographic, health, eyesight, and driving information to certify their validity for the study before starting, as well as to gather descriptive data and to characterize the independent variables (see “Main questionnaire” section). The inclusion criteria were: (1) not having been involved in similar tests; (2) having a visual acuity in distant sight of 0.6 or better in each eye, regardless of whether they used optical corrections; (3) in case of using optical corrections, this should be between -6 and +6 diopters both included; (4) not having oculomotor alterations, clinically significant crystalline opacification, nor health issues that may interfere with the reading or driving capacity; (6) not taking medication nor being involved in medical treatments that can interfere with reading or driving ability; (8) not having cognitive, neurological or ocular pathologies such as Alzheimer, Sclerosis; (9) not suffering from psychiatric disorders, nor having a history of substance abuse.

Once the eligibility of each participant was determined, they performed the ADEM test (after a thorough explanation), in order to obtain the outcomes for the dependent variables (see “ADEM test” section). The full session had a mean duration of 40 minutes. Potential biasing factors such as testing position and distance (sitting upright at a distance of 42.7±3.1cm from the test sheets), room lighting (always constant), and distracting factors (e.g., external noise or mobile phones) were controlled to avoid measurement gaps through rigorous surveillance of the research staff members.

#### Main questionnaire

The main questionnaire was designed following previously published questionnaires [28,42,43]. The questionnaire was anonymized through an alpha-numeric code. The paper-and-pencil collection method was used, and the questions were provided in Spanish. The instrument was composed of 33 items and structured in four sections (sociodemographic, health, eyesight, and driving information) as presented in the following section. It took approximately 10 minutes to complete.

*Sociodemographic information*. Age, gender, and academic level were inquired and used as demographic predictors in the linear model (see “Statistical analysis” section). The academic level was graded with an ordinal scale composed of three levels, 1 = Secondary education or lower, 2 = Middle (baccalaureate or equivalent), and 3 = University studies or equivalent.

*Health information*. Questions about health were extracted from previous ophthalmological research on elderly people [44]. The perception of one’s health has been recognized as a predictor of disability and it has been related to psychosocial and chronic health issues, including rheumatic, cardiovascular, or ophthalmic conditions [45–47]. Participants were inquired about their general health (suffered conditions such as diabetes, hypertension, thyroids, anemia, and others), involvement in drug treatment (muscle relaxants, antidepressants, sleep inducers), changes in diet, sleep, medication, trauma, or stress within the last three months. Moreover, participants were requested to rate their perceived quality of life on a Likert ordinal scale ranging from 0 to 4 (0 = very poor; 1 = poor; 2 = average; 3 = good; 4 = excellent).

*Eyesight-related information*. First, participants were asked about any suffered ocular condition, such as elevated intraocular pressure, strabismus, or ocular surgery (current or past). Afterward, they were asked about the quality they perceived in their vision and reading, also on a Likert ordinal scale (0 = very poor; 1 = poor; 2 = average; 3 = good; 4 = excellent). At this point, participants underwent the visual function index test VF-14 [48], which has already been validated in drivers [49]. This test consists of 14 questions related to the perceived difficulties in fourteen daily activities such as driving, reading, watching television, cooking. It has been strongly correlated with visual satisfaction, but not with visual acuity or health [48,50–53]. Answers were graded from 4 to 0, with 4 being “no difficulty” and 0 “unable to do it”. The final score was calculated by averaging all the answers and multiplying the mean by 25 in order to obtain a value out of 100. The Spanish version of the test was used, as it has been proved to be reliable, valid, and sensitive to change [54].

*Driving information*. In this section, participants were asked about their driving habits. The experience was defined as the years of being a driver. As there were no previous references, the experience was graded from 1 to 3 (0 = non-driver; 1 = less than 5 years; 2 = between 5 and 15 years; 3 = over 15 years). The frequency was estimated through the mean kilometers driven per week during the previous year, then extrapolated to kilometers driven per year. A scale was created, ranging from 1 to 3 (1 = under 2500km/year; 2 = between 2500 and 10000km/year; 3 = over 10000km/year). This value was used together with the experience to calculate the risk exposure on an ordinal scale (1 = very low risk, 2 = low risk, 3 = moderate risk, or 4 = high risk) as can be seen in Table 2. Also, a corrector factor was applied for the older groups, adding one point for those subjects above 65 and two points for those over 75. Furthermore, subjects rated the difficulty they perceived in day-and-night-driving (1 = none; 2 = little; 3 = moderate; 4 = much).

**Table 2:** Construction of risk exposure scale. A correction factor should be used for subjects over 65 years old.

	Low frequency	Medium frequency	High frequency
**Low-experienced**	Low risk	Moderate risk	High risk
**Medium-experienced**	Very low risk	Moderate risk	High risk
**High-experienced**	Very low risk	Low risk	Moderate risk

#### ADEM test

**Fig 2:**
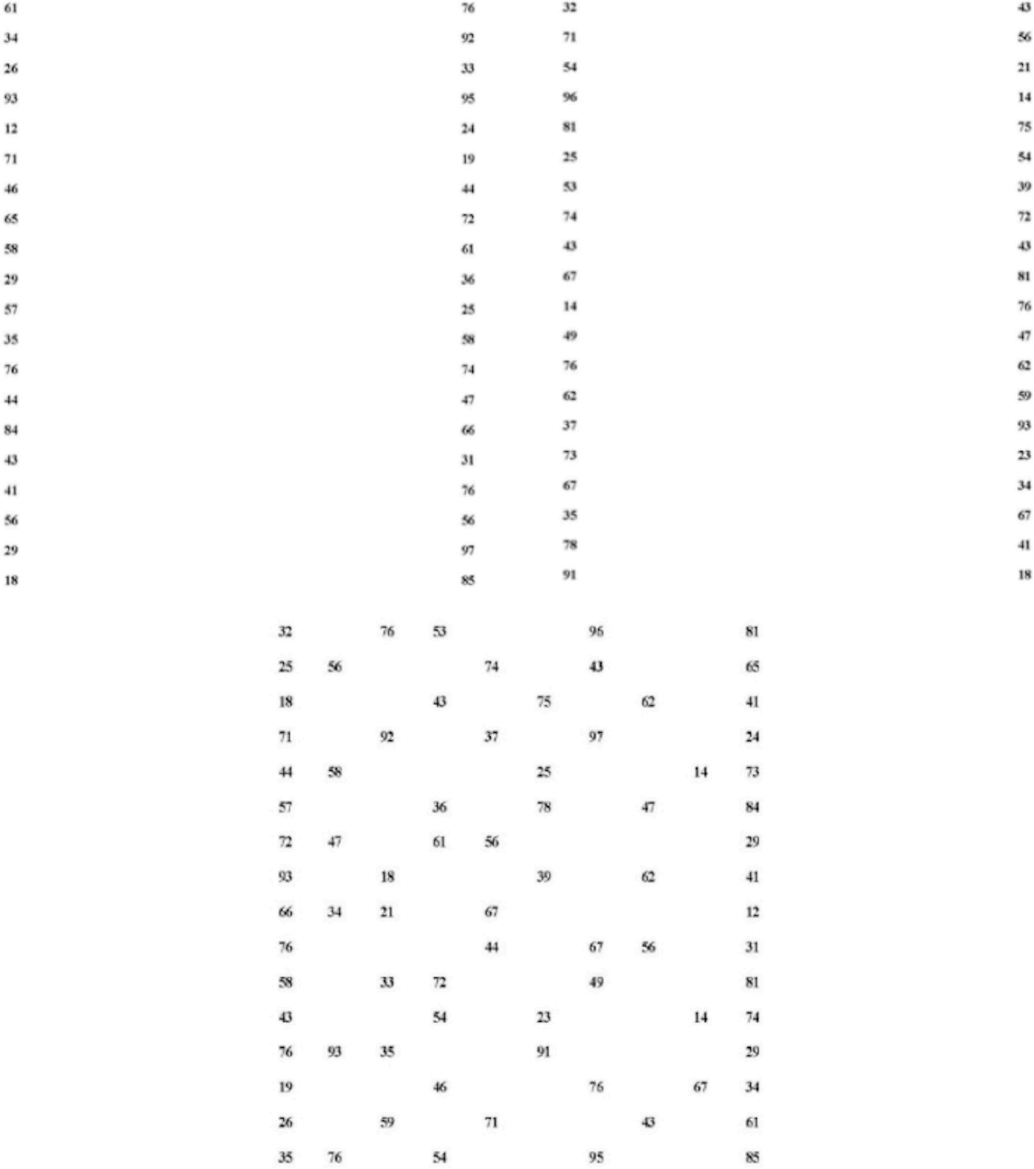
ADEM test vertical sheet 1 (upper left), vertical sheet 2 (upper right), and horizontal sheet (bottom).

The Adult Developmental Eye Movement test (ADEM) consists of three sheets of numbers, two containing 40 numbers vertically aligned and distributed in two columns, and one containing 80 numbers horizontally aligned and distributed in sixteen uneven rows (see Fig 2). It was developed in a Spanish-speaking population with ages between 14 and 68 years [20] and afterward validated in English-speaking subjects above 69 years old [14]. The test uses DIN-A4, size 11 Times New Roman letters equivalent to a Snellen resolution of 20/80 when presented at 40 centimeters.

The subjects had to read the numbers out loud as fast as possible, from top to bottom (vertical sheets) and from left to right (horizontal sheet). Participants were given the instruction not to stop when an error (omission or addition of numbers) occurred. A clinician recorded the speech with a tape recorder in order to evaluate the test performance ex-post, as explained below.

The test result is the time (in seconds) needed to read the sheets, which is adjusted by adding the errors (omissions or additions of numbers) in order to obtain different scores, as explained below. The time needed to read both vertical sheets (40 numbers each) was added up to obtain a single vertical value (time needed to read 80 numbers). Regarding the reliability, the first and the second vertical sheets showed a high consistency value (Spearman-Brown coefficient = 0.98). The reliability between the time needed to read the first forty numbers of the horizontal sheet and the time needed for reading the last forty numbers of the same horizontal sheet was 0.95. The scoring of the test was calculated considering the following guidelines.

1.- Adjusted vertical time (Vadj), which is a measure of the naming speed and automaticity: *Vadj (s) = Vx80/(80-omissions+additions)* where V (vertical time) are the seconds needed to read both vertical sheets.

2.- Adjusted horizontal times (Hadj), which is an indirect evaluation of pursuits and saccades combined with the automaticity in reading numbers and divided attention: *Hadj (s) = Hx80/(80-omissions+additions)* where H (horizontal time) are the seconds needed to read the horizontal sheet.

3.- As previously performed by Larter et al. [26] for the DEM test, the test ratio was calculated as *Hadj/Vadj*. In other words, the closer this ratio is to 1, the more proficient are the results obtained by the individual.

### Ethics

We conducted the study in conformity with the Code of Ethics of the World Medical Association (Declaration of Helsinki), and ethical approval was provided by the *Research Ethics Committee for Social Science in Health* of the INTRAS (University Research Institute on Traffic and Road Safety) of the University of Valencia (IRB approval number 000171016INT). All participants voluntarily agreed to participate; each participant signed an Informed Consent Statement and was free to withdraw from the study at any time. All the questionnaires and tests were designed and applied to ensure the anonymity of the participants. Data were confidential, and participation was anonymous, implying no potential risks for the integrity of the subjects.

### Statistical analysis (Data processing)

Statistical analyses were performed using commercial software (SPSS, Version 26.0). The normality of data distribution was assessed using the Kolmogorov-Smirnov test, showing a non-normal distribution. This fact indicates that test results did not follow a gaussian-bell-shaped distribution along with all the subjects. Bearing in mind the sample size, the homogeneity of variance was assessed through Levene’s test to check whether parametric tests could be carried out. However, homoscedasticity assumptions were not met. Thus, non-parametric Kruskal-Wallis for independent variables and Friedman for dependent variables were used to determine significant differences between groups (age, gender, academic level, and status of driver or non-driver) or between the ADEM sheets. To identify where the differences occurred, Mann-Whitney and Wilcoxon tests were used for independent and for dependent samples respectively.

At this point, Multiple Linear Regression analyses (MLR–method: Enter) were carried out for the dependent variables (Vadj and Hadj). Two models fit were tested as potential predictors of the vertical and horizontal adjusted times (Vadj and Hadj), one including sociodemographic (age, gender, and academic level) and one driving variables (experience, risk exposure, day and night driving difficulty). Gender was used as a “dummy” variable (category of success = being a male) in the prediction model. As mentioned above (see “driving information” section), non-drivers were included within the experience variable, enhancing the model interpretation and a succinct understanding of the analyses.

Moreover, Spearman (*rho*) correlation coefficient assessed the correlation between pairs of variables; this procedure was chosen considering its robustness over Pearson’s (*r*) coefficients when the study involves sets of variables measured using an ordinal scale.

The cut-off criteria or significance level for this study was uniformly established at *p* < .05. Reliability was tested through the Spearman-Brown correlation coefficient; values above 0.90 were considered of high measurement consistency.

## Results

### Temporal parameters of the sheets

Global results are shown below. It is important to bear in mind that the lower the time, the better the test performance. Table 3 shows the results of the test in all the sheets as raw (V and H) and adjusted (Vadj and Hadj) times. An average of an extra 5% time was needed to read the horizontal sheet in comparison with the vertical ones. The difference between the medians of the adjusted times for the vertical and horizontal sheets (Hadj-Vadj) was 3.2 seconds (*p* < .001). However, frequency analyses of the ratio Hadj/Vadj showed that 26.5% of the sample needed more time to complete the vertical sheet than the horizontal one, 4.3% needed the same time for both, and 69.2% needed more time for the horizontal sheet than for the vertical one. In this regard, a significant and strong correlation was found between Vadj and Hadj (*rho* = .900, *p* < .001).

**Table 3:** ADEM test global results.

	Range (secs)	Asymmetry	P25	Median	P75	Mean (SD)
**V**	30.0–171.0	1.7	51.0	58.0	73.6	64.31 (10.50)
**Vadj**	34.4–173.0	1.7	51.0	57.8	73.7	64.30 (20.68)
**H**	30.0–165.0	1.7	54.0	61.0	75.3	67.24 (20.80)
**Hadj**	30.0–165.0	1.7	54.0	61.0	75.3	67.55 (21.22)
**Hadj/Vadj**	.71–1.45	.29	.99	1.05	1.12	1.06 (.12)

*Notes for the table*: Values are expressed in seconds or points of asymmetry. Secs: seconds; P: percentiles; V: vertical sheets; H: horizontal sheet; adj: adjusted time; SD: standard deviation.

### Regression analyses

Multiple linear regressions were performed to predict the subjects’ test performance (vertical and horizontal adjusted times) based on their sociodemographic (age, gender, and academic level) and driving (experience, risk exposure, day and night driving difficulty) features. A significant regression equation was found for both test performances (Vadj: F(7,206) = 11.15, *p* < .001, with an adjusted R^2^ of .250; Hadj: F(7,206) = 12.32, *p* < .001, with an adjusted R^2^ of .271). Gender, academic level, risk exposure, and day-driving difficulty were discarded from the equation due to non-significant results. Participants’ predicted Vadj was equal to 47.145 + .503 (age) + 3.640 (night difficulty) - 6.225 (driving experience). Participants predicted Hadj was equal to 49.051 + .532 (age) + 4.240 (night difficulty) - 5.857 (driving experience). In both equations age is measured in years, night difficulty is coded from 1 to 4 (1 = none; 2 = little; 3 = moderate; 4 = much), and the driving experience is coded from 0 to 3 (0 = non-driver; 1 = less than 5 years; 2 = between 5 and 15 years; 3 = over 15 years). Regression analyses’ models are displayed in Table 4, where the significant model and its coefficients are described. Model 2 was retained, as it was the one with the greatest prediction potential. This model predicted 25% of the variance in the vertical times and 27% in the horizontal ones. Age, night driving difficulty, and driving experience were significant (*p* < .05) predictors of the test outcomes, with the age being the most robust one. As can be seen in the table, both age and night difficulty were positively correlated with the test times (worst test results), and the driving experience showed a negative correlation (best test results).

**Table 4:** Regression analyses.

**Model A—Dependent Variable: Vertical adjusted time**								
**Model**	**Predictor**	**Unstandardized Coefficients**		**Standardized Coefficients**	**t^(4)^**	**Sig. ^(5)^**	**Adj. *R*^*2*^^(6)^**	**△*R*^*2*^^(7)^**
		**B^(1)^**	**S.E.^(2)^**	**β^(3)^**				
1	(Constant)	39.916	4.522		8.827	.000	.168	.180
	Age	.370	.062	.424	5.978	.000		
	Gender	.360	2.077	.012	.173	.862		
	Educational level	.241	1.230	.013	.196	.845		
2*	(Constant)	47.145	6.578		7.167	.000	.250	.095
	Age	.503	.092	.577	5.453	.000		
	Gender	.920	2.026	.030	.454	.650		
	Educational level	-.248	1.187	-.013	-.209	.835		
	Experience	-6.225	2.430	-.265	-2.561	.011		
	Risk exposure	-1.405	1.102	-.084	-1.275	.204		
	Day difficulty	1.173	2.432	.033	.483	.630		
	Night difficulty	3.640	1.032	.241	3.527	.001		
**Model B—Dependent Variable: Horizontal adjusted time**								
**Model**	**Predictor**	**Unstandardized Coefficients**		**Standardized Coefficients**	**t**^**(4)**^	**Sig.** ^(5)^	**Adj. *R***^***2***^^(6)^	**△*R***^***2***^^(7)^
		**B**^**(1)**^	**S.E.**^**(2)**^	**β**^**(3)**^				
1	(Constant)	41.897	4.633		9.043	.000	.194	.205
	Age	.409	.063	.452	6.461	.000		
	Gender	.078	2.128	.002	.037	.971		
	Educational level	-.010	1.260	-.001	-.008	.994		
2*	(Constant)	49.051	6.747		7.270	.000	.271	.090
	Age	.532	.095	.587	5.625	.000		
	Gender	.568	2.078	.018	.273	.785		
	Educational level	-.504	1.217	-.026	-.414	.679		
	Experience	-5.857	2.493	-.240	-2.349	.020		
	Risk exposure	-1.009	1.130	-.058	-.893	.373		
	Day difficulty	-.921	2.494	-.025	-.369	.712		
	Night difficulty	4.240	1.058	.270	4.006	.000		

Notes for the table

*Retained model

;^(1)^B = Unstandardized effect coefficient

^(2)^S.E. = Standard Error

^(3)^β = Standardized effect coefficient (Beta–can be interpreted as controlling for the effects of other variables)

^(4)^*t =* Value of the Student’s *t*-test

^(5)^Sig = *p*-value of the test

^(6)^Adj. *R*^*2 =*^ Adjusted R-square

^(7)^△*R*^*2*^ = Changes in R-square.

### ADEM test results per age group

Table 5 shows the temporal outcomes obtained by the subjects for each sheet, divided by age groups. Results are presented as a median and IQR as a measure of dispersion for assessing the data´s homogeneity/heterogeneity. Moreover, the within-group Spearman correlation between vertical and horizontal times are shown as a performance parameter.

**Table 5:** ADEM test results divided by age.

Age group		≤ 24	25 to 34	35 to 44	45 to 54	55 to 64	65 to 74	≥75
**Vadj**	Median (IQR)	52.0 (7.0)	54.3 (14.6)	49.0 (14.1)	55.5 (17.8)	57.7 (23.6)	73.0 (30.4)	82.0 (38.2)
**Hadj**	Median (IQR)	55.5 (7.5)	57.0 (13.5)	54.0 (14.0)	61.5 (18.5)	65.0 (21.5)	69.1 (36.5)	92.1 (35.3)
**Corr. V-H**	*Rho* (*p-*value)	.61 (.001)	.90 (< .001)	.80 (< .001)	.84 (< .001)	.83 (< .001)	.92 (< .001)	.84 (< .001)
**Hadj/ Vadj**	Median (IQR)	1.06 (.13)	1.05 (.12)	1.03 (.10)	1.06 (.14)	1.06 (.15)	1.04 (.14)	1.06 (.18)

*Notes for the table*: Results are shown as the median and interquartile range (IQR) in seconds, and the Spearman correlation values (*Rho*). V: vertical sheets; adj: adjusted time; H: horizontal sheets.

### Influence of the driver status

Table 6 shows the results of the ADEM divided by drivers and non-drivers. These results are in line with those obtained in the linear regression analyses, in which the driving experience (including not being a driver) was found to be a potential predictor of the test performance.

**Table 6:** ADEM test results divided by group drivers vs. non-drivers.

Status	Vadj	Hadj	Hadj/Vadj
Driver	54.7 (15.6)	58.1 (14.1)	1.05 (.13)
Non-driver	75.0 (35.1)	75.5 (35.1)	1.05 (.14)
*p*-value	< .001*	< .001*	.64

*Notes for the table*: Results are shown as the median and interquartile range (IQR) and in seconds.

* Statistically significant difference at *p* < .001 level; V: vertical sheets; adj: adjusted time; H: horizontal sheets.

In addition to what was found in the regression analyses (predictive value of experience and night difficulty), and although the risk exposure (calculated computing frequency and experience) was excluded from the regression analyses, it is worth highlighting that the kilometers driven per year (frequency) showed a significant and negative association with the test results in both the vertical (*rho* = -.206, *p* = .002) and horizontal adjusted times (*rho* = -.164, *p* = .017); which means that the more kilometers driven per year, the better test performance.

## Discussion

This study proposes the use of a simple visual-verbal test that characterizes the parameters of cognitive and visual attention that could be important in driving performance. Bearing in mind the purpose of the study, this investigation examined the results of 302 subjects (drivers and non-drivers) in the Adult Developmental Eye Movement Test (ADEM). This test could be of interest for detecting certain visual processes and/or cognitive automaticity deficiencies in drivers during their eyesight examination, necessary to obtain or renew the driving license [3,27,28]. The importance of this evaluation could also lie in the relevance of understanding the drivers’ visual strategies and processes, used for determining their visual information management and predicting their driving performance [5,7,17]. The most notable findings were that drivers’ testing times were lower than non-drivers’ (see Table 6), which confirms the first hypothesis. And also, that the age, followed by the driving experience, and the perceived difficulty in driving at night were the main test performance predictors.

For the development of this discussion, we will first analyze the temporal parameters and structure of the ADEM. Second, we will address the potential influence of the independent variables that were analyzed (driving and sociodemographic parameters). It is worth mentioning that this is the first study discussing this matter and, thus, comparisons should be made with caution.

### ADEM parameters

Results of the general sample can be found in Table 3. Concisely, more time was required to complete the horizontal sheet compared to the sum of the vertical sheets. This could be confusing, as horizontal eye-movements are far more frequent in reading and driving tasks [15]. However, we believe that our results may be conditioned by the difference in the numbers spatial arrangement between both sheets (see Fig 2). Regarding this point, both studies that used the ADEM in their methods reported a higher horizontal (Hadj) time compared to the vertical (Vadj) one, due to their higher complexity [14,20]. In addition, a significant and strong correlation was found in our study between Vadj and Hadj times (*rho* = .900, *p* < .001), as reported by Gene-Sampedro and colleagues (*r* = .90 *p* < .001; [20]). It is also worth mentioning that the ratio Hadj/Vadj was found to be stable in the influence of the analyzed independent variables. One of the uses of the test may be to detect subjects that obtain bad results in any test parameters, compared to the population’s values [20]. Since most people have optimal results, this may be helpful for detecting impairments in any of the constructs that the test measures (oculomotor function, processing, and/or attention).

### Influence of driving variables in test performance

As previously mentioned in the discussion, drivers performed the test with better results than non-drivers. Bearing in mind the second hypothesis, an effect of the driving experience (as a predictor in the regression model) and frequency (as a negative correlation) in the test performance of our study was observed. With both parameters being associated with better test performance. In other words, the more years spent driving, and the more kilometers driven per year, the better results obtained in the test. This reinforces the hypothesis of drivers gaining certain oculomotor and cognitive training while performing the driving task [28]. Accordingly, in a study comparing the carryover effect of eye-movements between two tasks, it was found that non-drivers had worse results than drivers, and that novel drivers performed worse than experienced drivers [15]. In this sense, experienced drivers were reported to use overt (with fixations) and covert (without fixations) attentional strategies better than non-drivers or novice drivers to detect hazards [15]. Also, it has been reported that inexperienced drivers are more likely to focus on central vision [3] and have a longer fixation duration in many situations [28].

Regarding the frequency, a poor but significant negative correlation with age was found (*rho* = -.138 p = .043); this implies that the older the subject, the fewer the kilometers driven. Bearing in mind that age is the main independent variable acting as a predictor of test performance (see Table 4), this could modulate the correlation between the kilometers driven per year (frequency) and the test performance. It would also explain why the risk exposure (calculated as a ratio between the frequency and the experience) was excluded from the regression model.

Finally, a higher night-driving perceived-difficulty was found to be a significant predictor of worse test performance. This may be linked to visual deficiencies that make the visual task difficult when driving under low light conditions [10], and that could not be detected in basic eyesight examinations, such as those carried out in our study when certifying the validity of participants to join the research. In this context, Cestac et al. [12] pointed out that the driver’s perceived ability to manage the driving task in accordance with its perceived difficulty increases with experience so that the group of experienced drivers can compensate for potential unfavorable situations.

### Sociodemographic variables that influence test performance

Differences in the test performance between age groups (see Table 5) were observed, thus supporting the second hypothesis. While age was the most powerful predictor of the test performance in the adopted regression model (see Table 4), the educational level and the gender of participants showed non-significant prediction values. These data provide some support to the application of the test, highlighting the necessity of considering age as a potential influencer of the test performance. No influence of gender was detected in the test outcomes. Some interesting discussion on the potential relationship between the academic level of the subjects and the test results are displayed further below.

Our results match those presented by Gene-Sampedro et al. [20] in terms of test performance among different age groups and showed that younger subjects (under 44 years old) had better results than older ones in almost all the testing variables that were analyzed. Those results are consistent with other authors who found a decline in ocular searching speed with the increase of age [6,27,28]. Accordingly, Guidetti et al. [6] concluded that age below 30 seems to guarantee a better precision of performance, as well as accuracy in detecting visual targets through saccadic eye movements. Furthermore, our results became unstable and more differences were found when assessing older age groups. This too could be related to the influence of age in ocular movements during the reading and driving processes [14,27,28,55], with older people needing more fixation time and having slower saccades needed to follow the same scene [4,6,20]. Although our study does not consider driving behaviors, it is important to note that, taking into account the available evidence on the matter, more positive attitudes are usually observed in middle-aged drivers, while young people aged between 18 and 25 years old are more exposed to driving violations and personality-related risk factors [32,56]. Also, elderly groups of drivers are considered a dangerous group, due to their slower visual processing speed under divided attention conditions [27,28,34]. In the same way, one study concluded that the most positive attitudes are found in ages between 31 and 40 years old [29]. Also, our data showed that test times begin worsening after the age of 45, being the group between 35 and 44 years old the one with the best test results (see Table 5). This may be due to the neurological evidence showing that the brain development for inhibition and control is not complete until 25 years of age [57]. Understanding human development stages [57,58], this may imply that participants younger than this age (and older ones, due to the aging processes) may have worse capacities for the test performance than middle-aged subjects.

Concerning gender, it can be said that it is not a potential predictor of the performance in the ADEM and, therefore, this test does not report a higher prevalence of visual issues in females when compared to males, as previously reported [10].

It is worth highlighting that the academic level of the subjects was not a potential predictor of the test performance, although significant differences were found in test performances between the basic educational level and both the medium (*p* < .05) and the high levels (*p* < .001), but not between the medium and the high level (*p*>.05). Our results could imply that people with higher levels of academic training had better results in test times, which may be related to factors such as further visual impairment coming with the aging of people in socioeconomic disadvantage [59]. However, the educational level of subjects was excluded from the regression model due to not presenting statistically significant values. This is probably due to the existing significant negative correlation (*rho* = -.345, *p* < .001) between educational level and age. Considering age as the main predictor of the test performance (see “*Regression analyses*” section), this abovementioned correlation may modulate the effect of the academic level in the test performance, as it would explain why the academic level was excluded from the regression model.

### Limitations of the study and future research

Although our sample size was considerably large, and all statistical parameters were accurately and positively tested during the treatment of our data, some specific issues or biasing sources should be acknowledged.

First of all, and although our results are interesting and significant, further research on the validity of this test in the context of drivers’ eyesight examination should be carried out. Also, self-report-based methods were used to gather data on the independent variables, and several studies have shown how self-report measures may imply different biases, such as acquiescent answers (i.e., the total agreement of participants with the presented questions), social desirability, and lack of sincerity. Furthermore, positive/negative affects/mood may impact the response style of participants, especially when addressing issues that may seem sensitive, even when responding to anonymous questionnaires, as pointed out by Chai et al. [60] and Af Wåhlberg [61] in previous studies dealing with drivers. However, these methods were used for the independent variables, and the dependent variables were obtained through direct testing procedures.

Second, and regarding the conditions of the assessment test, we should say that it was not performed within the usual driving environment context, where the objects that have to be perceived are rarely stationary but rather move at different speeds and in different directions around the subject. But drivers are indeed constantly moving their eyes and head in such an environment, in order to catch and locate the images in their retina. In this regard, a visual tracking device could have been used to evaluate saccadic eye movements in combination with the reading test. Also, it is important to point out that future research should be developed in order to further validate the use of ADEM in drivers. No previous research has evaluated the test-retest intersession reliability, and thus our results in this regard are solely comparable with the DEM results, which has some reliability controversy.

Third, regarding the sample, the population of this study only includes Caucasian individuals, so that the results are limited and cannot be generalized to other ethnicities. Also, as previous research has pointed out, it is important to bear in mind the language barriers that can affect the performance of the test [14].

Finally, previous literature has indicated percentiles and adjustments in age for what concerns the time results of the ADEM test [20]. As our results showed, it would be interesting to adjust the test outcomes depending on driving parameters as well. Further research should investigate other sources of bias that may condition the performance in this visual-verbal test. For instance, it would be of great interest to try to predict the test performance through the road crashes suffered by subjects, or to relate the test results with road hazard detection in a similar way as done in the studies by Hills et al. [15,16].

## Conclusion

In conclusion, and bearing in mind all the aforementioned facts, our results showed that drivers have better performance in the assessed visual-verbal test than non-drivers. Moreover, certain demographic (age) and driving (experience, perceived difficulty at night) parameters act as potential predictors of the test performance. The ADEM test could be a useful, inexpensive tool for eyesight examinations in driving recognition centers. While this visual-verbal test may not directly assess ocular movements or driving performance, it may be helpful in indirectly detecting oculomotor function and visual-verbal attentional issues, which may be related to a worsened driving performance.

**Table:** Annex 1: Table of abbreviations.

Abbr	Meaning	Brief description
ADEM	Adult Developmental Eye Movement Test	Visual test which examines the ocular movements performed while reading.
Adj	Adjusted time	Recalculated times considering errors.
H	Horizontal sheet	Horizontal sheet of the ADEM test.
HLC	Horizontal level of competence	Recalculated times considering errors and scaled from 1 to 5. The higher the grade the better the performance.
IQR	Interquartile Range	Measure of statistical dispersion calculated through the difference between 75th and 25th percentiles, or between upper and lower quartiles.
QHTP	Quality of horizontal test performance	Percentages of the performance in the test times considering errors. The higher the ratio, the higher the quality of the test performance.
QVTP	Quality of vertical test performance	
SD	Standard Deviation	Measure of the amount of variation or dispersion of a set of values.
UFOV	Useful Field of View	Predictor tests for a range of driving outcomes measures, including driving ability and crash risk as well as other everyday tasks.
V	Vertical sheet	Vertical sheet of the ADEM test.
VF14	Visual Function Index Test	Brief questionnaire designed to measure functional impairment in patients, caused by cataract.
VLC	Vertical level of competence	Recalculated times considering errors and scaled from 1 to 5. The higher the grade the better the performance.

## Supporting information

S1 Appendix Questionnaire used to obtain sociodemographic information, general and visual health data (including VF14) and driving characteristics are fully available in this file. (DOCX)

S2 Appendix Reference population time needed to assess level of competence (Retrieved from: Gene-Sampedro A, Richman JE, Pardo MS. The Adult Developmental Eye Movement Test (ADEM) a tool for saccadic evaluation in adults. J Behav Optom. 2003;14(4):101–5.) are fully available in this file.
(PDF)

S1 Raw dataset Raw data is available in the file (database) attached to the electronic version of this manuscript. (SAV)
