# Supplementary material for: Comparing oculomotor efficiency and visual attention between drivers and non-drivers through the Adult Developmental Eye Movement (ADEM) test: A visual-verbal test
Source: PLoS One. 2021 Feb 5;16(2):e0246606. doi: 10.1371/journal.pone.0246606 (PMC7864424; doi:10.1371/journal.pone.0246606)
Supplement: S1 Appendix — (DOCX) [file pone.0246606.s001.docx]

**S1 Appendix. Questionnaire contents (root - researcher form)**

**Questionnaire code: _________**

*How to fill out the questionnaire? The questionnaire is divided in two sections, that you will have to answer in the indicated spaces. In case of a checkbox, select the one that better corresponds to you through a cross X. If you don´t know the answer to a question, don´t worry, you may skip it.*

**General information**

Date of birth: __/__/____

Male/man Woman/female

*Educational level*: High (university), medium (high school), low (middle and primary school)

*Do you habitually drive a car?* Yes/no

Average km driven per week: ______ km.

*Have you had any driving accident, while you were driving, during the past 3 years?*

No Yes (not severe) Yes (severe)

*Generally speaking, how would you assess your quality of life?* (please use a vertical mark on the line below)

Awful/bad/normal/good/excellent

*In the past 3 months, have you experienced any changes in…?*:

Diet, Sleep patterns, Medication, Trauma, Stress

*General health: Any problem(s) of*: Diabetes, Hypertension, Thyroid, Anemia, Others

*Medication. Are you habitually taking?:*

Muscular Relaxant, Antidepressants, Sleep medication

**Visual information**

*Ocular treatment (on-going or past*): Ocular Tension, Lazy Eye, Strabismus, Surgery

*Generally speaking, how would you consider your quality of sight* (if you use glasses, with them)? Excellent, Good, Normal, Bad, Awful

Does your sight worsen during nighttime? Yes No

*Do you habitually read?* (journals/magazines, news, books, on the computer, etc) Yes No

If your answer was yes, how much weekly time do you usually dedicate to these activities?

Less than 3 hours/ from 3 to 6 hours/ from 7 to 14 hours/ from 15 to 21 hours/ more than 21 hours

*Generally speaking, how would you consider your quality of reading?*

Excellent, Good, Normal, Good, Awful

*How difficult is driving for you, because of your sight?*

During the day: I don’t drive/ None/ A little bit / Moderate/ Very difficult

During the night: I don’t drive/ None/ A little bit / Moderate/ Very difficult

**Questionnaire to assess functional visual abilities VF-14**

Possible Answers: Not applicable/ no / a little bit / moderate/ a lot / I cannot do it

*Do you experience any difficulties, even with glasses, when reading a small printed text, such as medication label, a telephone list, or a food label?*

*Do you experience any difficulties, even with glasses, when reading a book or newspaper?*

*Do you experience any difficulties, even with glasses, when reading a book with big font, or a big text in a newspaper, or a telephone number?*

*Do experience any difficulties, even with glasses, when recognizing people who approach you?*

*Do you experience any difficulties, even with glasses, when seeing curbs, stairs or slopes?*

*Do you experience any difficulties, even with glasses, when reading traffic signs/signals, street names, or shop signs?*

*Do you experience any difficulties, even with glasses, when performing manual tasks, such as sewing, embroidery, crochet, carpentry?*

*Do you experience any difficulties, even with glasses, when writing down or filling out forms?*

*Do you experience any difficulties, even with glasses, when playing bingo, domino, card games etc.?*

*Do you experience any difficulties, even with glasses, when taking part in sports such as golf, tennis, handball, bowling etc.?*

*Do you experience any difficulties, even with glasses, when cooking?*

*Do you experience any difficulties, even with glasses, when watching television?*

**Driving-related visual information**

*Do you habitually drive a car?* Yes No

How many km do you drive, on average, per week?

*How much difficulty do you experience when driving by night, because of your sight?*

None/ A little bit / Moderate/ A lot

*How much difficulty do you experience when driving by day, because of your sight?*

None / A little bit / Moderate / A lot

*How frequently do you use a car?*

Sporadically/ Sometimes / Moderately / Often
