# Supplementary material for: Comparing oculomotor efficiency and visual attention between drivers and non-drivers through the Adult Developmental Eye Movement (ADEM) test: A visual-verbal test
Source: PLoS One. 2021 Feb 5;16(2):e0246606. doi: 10.1371/journal.pone.0246606 (PMC7864424; doi:10.1371/journal.pone.0246606)
Supplement: S2 Appendix — The Adult Developmental Eye Movement Test (ADEM) a tool for saccadic evaluation in adults. J Behav Optom. 2003;14(4):101–5.) are fully available in this file. (PDF) [file pone.0246606.s002.pdf]

Age Ranges in Subject Goups		
Group	Age range (years)	N
1	14-18	48
2	19-23	52
3	24-28	60
4	29-33	45
5	34-38	45
6	39-43	45
7	44-48	45
8	49-53	45
9	54-58	45
10	59-63	45
11	64-68	45

Means (SD) for Vertical Score Time		
Group	Vertical time (secs)	
	Mean	±SD
1	50.50	8.64
2	45.23	6.60
3	44.93	7.22
4	47.43	7.46
5	47.18	7.88
6	51.24	7.64
7	54.58	10.01
8	57.64	11.18
9	57.04	12.46
10	64.37	8.43
11	66.00	10.44

Means (SD) for Horizontal Score Time		
Group	Horizontal time (secs)	
	Mean	±SD
1	51.21	12.85
2	49.93	8.23
3	47.63	7.32
4	48.61	7.73
5	50.09	10.47
6	51.34	6.92
7	56.80	10.78
8	57.82	9.45
9	58.01	10.95
10	65.18	10.87
11	69.46	10.14

Means (SD) for Horizontal/Vertical RatioScore		
Group	Ratio	
	Mean	±SD
1	1.01	0.10
2	1.08	0.12
3	1.06	0.13
4	1.03	0.11
5	1.06	0.11
6	1.01	0.09
7	1.05	0.11
8	1.01	0.11
9	1.03	0.10
10	1.01	0.08
11	1.06	0.10
